# Supplementary material for: Novel Driver Strength Index highlights important cancer genes in TCGA PanCanAtlas patients
Source: PeerJ. 2022 Aug 11;10:e13860. doi: 10.7717/peerj.13860 (PMC9375969; doi:10.7717/peerj.13860)
Supplement: Supplemental Information 2 [file peerj-10-13860-s002.zip › COHORTS/genes plots/age/2021_8_16_14_9_distribution_cohorts_females_55-59.pdf]

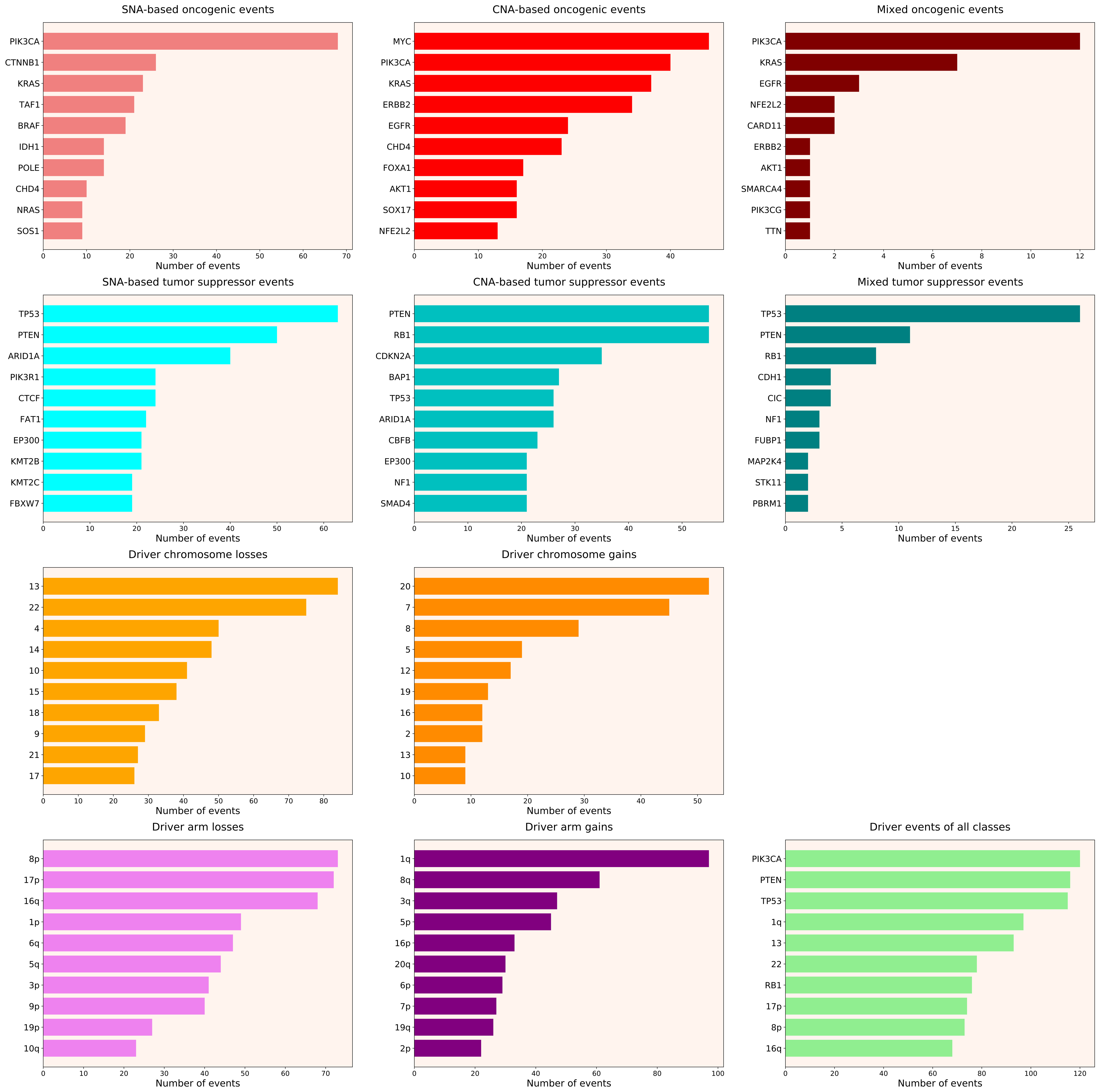
SNA-based oncogenic events
PIK3CA
CTNNB1
KRAS
TAF1
BRAF
IDH1
POLE
CHD4
NRAS
SOS1
0 10 20 30 40 50 60 70
Number of events
CNA-based oncogenic events
MYC
PIK3CA
KRAS
ERBB2
EGFR
CHD4
FOXA1
AKT1
SOX17
NFE2L2
0 10 20 30 40
Number of events
Mixed oncogenic events
PIK3CA
KRAS
EGFR
NFE2L2
CARD11
ERBB2
AKT1
SMARCA4
PIK3CG
TTN
0 2 4 6 8 10 12
Number of events
SNA-based tumor suppressor events
TP53
PTEN
ARID1A
PIK3R1
CTCF
FAT1
EP300
KMT2B
KMT2C
FBXW7
0 10 20 30 40 50 60
Number of events
CNA-based tumor suppressor events
PTEN
RB1
CDKN2A
BAP1
TP53
ARID1A
CBFB
EP300
NF1
SMAD4
0 10 20 30 40 50
Number of events
Mixed tumor suppressor events
TP53
PTEN
RB1
CDH1
CIC
NF1
FUBP1
MAP2K4
STK11
PBRM1
0 5 10 15 20 25
Number of events
Driver chromosome losses
13
22
4
14
10
15
18
9
21
17
0 10 20 30 40 50 60 70 80
Number of events
Driver chromosome gains
20
7
8
5
12
19
16
2
13
10
0 10 20 30 40 50
Number of events
Driver arm losses
8p
17p
16q
1p
6q
5q
3p
9p
19p
10q
0 10 20 30 40 50 60 70
Number of events
Driver arm gains
1q
8q
3q
5p
16p
20q
6p
7p
19q
2p
0 20 40 60 80 100
Number of events
Driver events of all classes
PIK3CA
PTEN
TP53
1q
13
22
RB1
17p
8p
16q
0 20 40 60 80 100 120
Number of events
